# Direct Growth of Low Thermal Conductivity WTe_2_ Nanocrystalline Films on W Films

**DOI:** 10.3390/nano14050401

**Published:** 2024-02-22

**Authors:** Zhisong Yu, Rong Tao, Jin Guo, Shiyi Feng, Yue Wang

**Affiliations:** School of Physical Science and Technology, Inner Mongolia University, Hohhot 010021, China

**Keywords:** WTe_2_, direct growth, thermal conductivity

## Abstract

WTe_2_ has attracted much attention because of its layered structure and special electronic energy band structure. However, due to the difficulty of evaporating the W element itself and the inactivity of the Te element, the obtained large-area WTe_2_ thin films are usually accompanied by many defects. In this paper, WTe_2_ nanocrystalline films were successfully prepared on quartz substrates using magnetron sputtering and chemical vapor deposition techniques. Various analytical techniques such as X-ray Diffraction, Raman spectra, X-ray Photoelectron Spectroscopy, Scanning Electron Microscope, and photoluminescence spectra are employed to analyze the crystal structure, composition, and morphology. The effects of different tellurization temperatures and tellurization times on the properties of WTe_2_ thin films were investigated. WTe_2_ nanocrystalline films with good crystallinity were obtained at 600 °C for 30 min. The thermal conductivity of the WTe_2_ films prepared under this condition was 1.173 Wm^−1^K^−1^ at 300 K, which is significantly higher than that of samples prepared using other methods.

## 1. Introduction

Two-dimensional transition metal dichalcogenides (TMDCs) exhibit various physical properties, including high electrical conductivity, high thermal conductivity, and high optical transparency. Among them, WTe_2_, as a typical TMDs material, exhibits low thermal conductivity due to its special crystal structure and phonon transport properties. It has a wide range of applications in the fields of electronic devices, thermoelectric materials, and energy storage [1,2,3,4]. In addition, WTe2 is characterized by unsaturated giant magnetoresistance, narrow bandgap and high pressure superconductivity [5,6,7]. It can be applied in field effect transistors and energy storage devices [8,9,10]. However, to fully utilize the potential of WTe_2_ in practical applications, the issue of its controllable growth on various substrates needs to be addressed first. In the field of micro- and nanoelectronics, tungsten (W) film is a commonly utilized metal film known for its excellent electrical conductivity and stability, making it a crucial component in various electronic devices. If WTe_2_ nanocrystalline films can be grown directly on W films, it will provide new possibilities for the preparation and application of WTe_2_-based composites. WTe_2_ has long been recognized for its good thermoelectric properties, and WTe_2_ films with low thermal conductivity can be used to prepare highly efficient thermal isolation layers that can help improve the performance and stability of electronic devices. In addition, such WTe_2_ films can be utilized in thermal management technologies to mitigate heat loss and enhance the energy efficiency of electronic devices. The availability of lower thermal conductivity WTe_2_ films gives rise to more potential for its use in electronic devices. However, the inactivity of tellurium makes it more challenging to prepare WTe_2_ films, making industrial preparation crucial for their next production applications [11,12,13,14,15,16]. Although there have been some studies on WTe_2_, there have been relatively few technical studies on the direct growth of WTe_2_ on W thin films. Up to now, there are still imperfections in the methods used to prepare WTe_2_ thin films. These include the poor crystallinity of the films obtained through the one-step method of CVD, the uncontrollable thickness of the films in the two-step method, and other issues [17,18,19,20,21]. There is a lack of a cost-effective method for the preparation of tunable synthesis of a large number of small-sized tungsten telluride thin films to meet the specific needs of thin film materials in terms of size and stability for specific applications such as luminescence, thermal conductivity, and photocatalysis [22]. This greatly limits the wide application of WTe_2_ in practical settings. Therefore, it is of great theoretical significance and practical application value to systematically study the direct growth technology of WTe_2_ nanocrystalline films with low thermal conductivity on W films and to explore the effects of growth conditions and microstructure on the properties of WTe_2_ films.

The magnetron sputtering process has the advantages of self-limitation, excellent three-dimensional shape preservation, large-area uniformity, and accurate film thickness control [23,24,25], which makes it an efficient method for the preparation of large-area WTe_2_. However, due to the inactivity of elemental tellurium, the growth of WTe_2_ directly on magnetron-sputtered tungsten films is very challenging and has seldom been reported. We obtained by a new experimental approach that a homogeneous disordered nanorod-like morphology may be an efficient path for growing WTe_2_ films with lower thermal conductivity. In this paper, WTe_2_ was successfully grown directly on a tungsten film in a tube furnace by adding hydrogen to aid growth, using tellurium powder as the raw material, and optimizing the growth temperature and time. The composition and structure were also confirmed through a series of characterizations. In the meanwhile, research on the effects of tellurization temperature and tellurization time on tungsten telluride films yielded the ideal growth conditions for WTe_2_ nanocrystalline films. It was found that the films grown under the conditions yielding the best film quality exhibited a nanorod-like structure. We have also examined the properties of this nanocrystalline film, which was discovered to have fewer lattice defects through photoluminescence spectroscopy under optimal experimental conditions. In addition, the thermal conductivity of the samples grown by this method was found to be significantly lower than those prepared by other methods, as determined by thermal testing. This may be due to the uniformly disordered rod-like structure of the WTe_2_ films grown using our method.

## 2. Experiment

W films were deposited on quartz glass substrates using a magnetron sputtering system, a triple-target magnetron sputterer VTC-600-3HD from Shenyang Kejing Auto-instrument Co., Ltd. (Shenyang, China). The deposition thickness was controlled by a thickness monitor in combination with the exposure time. The molecular pump was turned on to lower the pressure of the vacuum chamber to below 10^−5^ Pa before sputtering, and the substrate was heated to 100 °C. After that, high-purity argon gas was introduced into the chamber, and the pressure inside the vacuum chamber reached the glow pressure. The DC power supply was switched on for the glow process. Once the glow is established, the speed of the molecular pump and the flow of argon was adjusted to control the pressure inside the chamber until it reaches the desired working pressure. After reaching the working pressure of 1 Pa, the DC power supply was adjusted to a deposition power of 40 W. The power of the DC power supply was adjusted to the deposition power of 40 W. The thickness of the deposited precursor tungsten film was 30 nm. The morphology of the deposited precursor tungsten films was relatively flat, which has a minimal influence on the study of the morphology of the subsequent WTe_2_ films. A three-temperature tube furnace, OTF-1200 X from Hefei Kejing Materials Technology Co., Ltd. (Hefei, China), was utilized for the tellurization of tungsten films.

**Figure 1:**
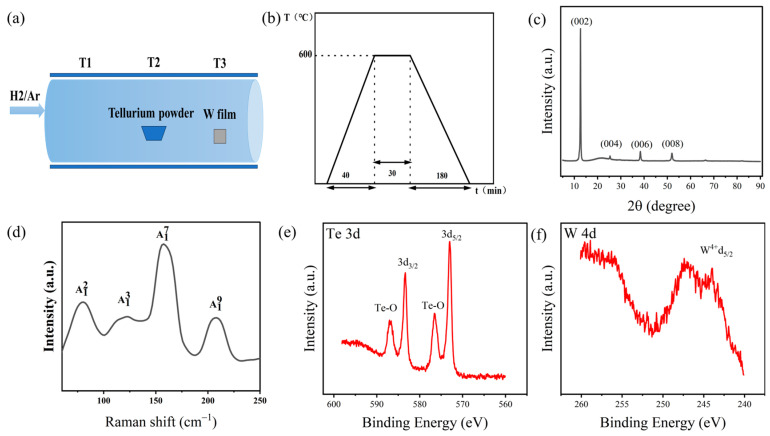
(**a**) Schematic diagram of the WTe_2_ preparation apparatus; (**b**) WTe2 rise and fall temperature curve; (**c**) XRD pattern; (**d**) Raman spectrum; (**e**) Te 3d XPS spectrum; (**f**) W 4d XPS spectrum.

The tungsten precursor film was placed in the T3 temperature region, as illustrated in Figure 1a. One gram of high-purity tellurium powder was weighed into the T2 temperature zone. The tube was evacuated to 1 Pa using a mechanical pump and then filled with high-purity argon gas three times to wash the gas. Finally, a mixture of argon and hydrogen containing 10% hydrogen was introduced as a carrier gas to return the tube to atmospheric pressure. With the outlet valve open to maintain a carrier gas flow rate of 100 sccm. The tube furnace was started and the T2 and T3 temperature zones of the tube furnace were heated up to 600 °C at a rate of 15 °C per minute and maintained at this temperature for 30 min. The temperature rise and fall curves were shown in Figure 1b. The temperature was naturally decreased to room temperature at the end of the reaction.

X-ray photoelectron spectroscopy (XPS) was performed using an ESCALAB 250Xi X-ray photoelectron spectrometer manufactured by Thermo Scientific, Waltham, MA, USA. X-ray diffraction (XRD) was measured using a D8 ADVNCE diffractometer from Bruker, Saarbrücken, Germany. Raman tests were performed with a LabRAM HR evolution from Horiba scientific, Kyoto, Japan. A scanning electron microscope (SEM) using a Mira4 from Tescan, Brno, Czech Republic, was used to visualize the morphology. Thermal conductivity was tested using a NETZSCH-LFA 467HT Laser Thermal Conductivity Tester from NETZSCH-Gerätebau GmbH, Selb, Germany. Photoluminescence analysis (PL) was performed with a spectral analyzer HAAS-2000 from Hangzhou Yuanfang Company (Hangzhou, China).

## 3. Results and Discussion

In order to understand the material composition of the grown films. We investigated the structural composition of the films using X-ray diffraction, X-ray photoelectron spectroscopy, and Raman spectroscopy. Figure 1c shows the XRD pattern of WTe_2_ prepared at a reaction temperature of 600 °C and a reaction time of 30 min. The main diffraction peaks of the pattern are in high agreement with the standard PDF card of WTe_2_ (ICSD No. 71-2156) (The Inorganic Crystal Structure Database). The strongest diffraction peak occurs at 2θ = 12.6°, corresponding to the (0 0 2) crystal plane. Diffraction peaks were also detected for (0 0 4), (0 0 6), and (0 0 8) crystal planes, all of which are (0 0 2n) (n = 1, 2, 3, …) crystal planes. It indicates that the tungsten ditelluride thin films prepared by the two-step method are well-oriented. Figure 1d shows the Raman spectral image of the WTe_2_ thin film obtained using a laser with an excitation wavelength of 532 nm. The Raman spectrum shows that there are four distinct Raman peaks of WTe_2_ in the wavelength range of 50–250 cm^−1^, which are located at 80.5 cm^−1^, 122.7 cm^−1^, 157.5 cm^−1^, and 208.4 cm^−1^, corresponding to the $A_{1}^{2}$, $A_{1}^{3}$, $A_{1}^{7}$, and $A_{1}^{9}$ of the W–Te bonding vibrational modes. Among them, $A_{1}^{2}$, $A_{1}^{3}$, and $A_{1}^{9}$ belong to in-plane vibrations, while $A_{1}^{7}$ belongs to out-of-plane vibrations [26]. The two strongest Raman peaks located at 157.5 cm^−1^ and 208.4 cm^−1^ can be taken as the characteristic peaks of WTe_2_, which proves once again that the generated films are WTe_2_.

Figure 1e shows the XPS scanning spectrum of Te 3d. The peak binding energies of Te 3d_5/2_ and 3d_3/2_ are 573 eV and 583.35 eV, respectively. The peaks at 586.65 eV and 576.55 eV belong to the Te–O bonding, suggesting that there is a small portion of tellurium oxide in the film. Figure 1f shows the appearance of the W 4d_5/2_ peak at a binding energy 244.5 eV for W 4d. This indicates the formation of a W–Te bond and confirms the success of the second step of the tellurization reaction. The content of O1s was analyzed using an XPS full spectrum and accounted for 27.61% of the full spectrum. It is possible that WOx or other oxides were generated, but the XRD shows mainly WTe_2_ peaks and no WOx peaks, which means that the WOx content is not high. The amount of O 1s may be due to the fact that XPS only scanned the surface, which may have been oxidized during transport, and some O 1s peaks appeared.

Through the various characterization methods mentioned above, we have demonstrated that we successfully prepared high-quality Td phase tungsten ditelluride thin films by a two-step method. There are many factors that affect the quality of the films during the preparation process, such as reaction time and reaction temperature. Therefore, we investigated and optimized the effects of these two conditions on the film quality.

**Figure 2:**
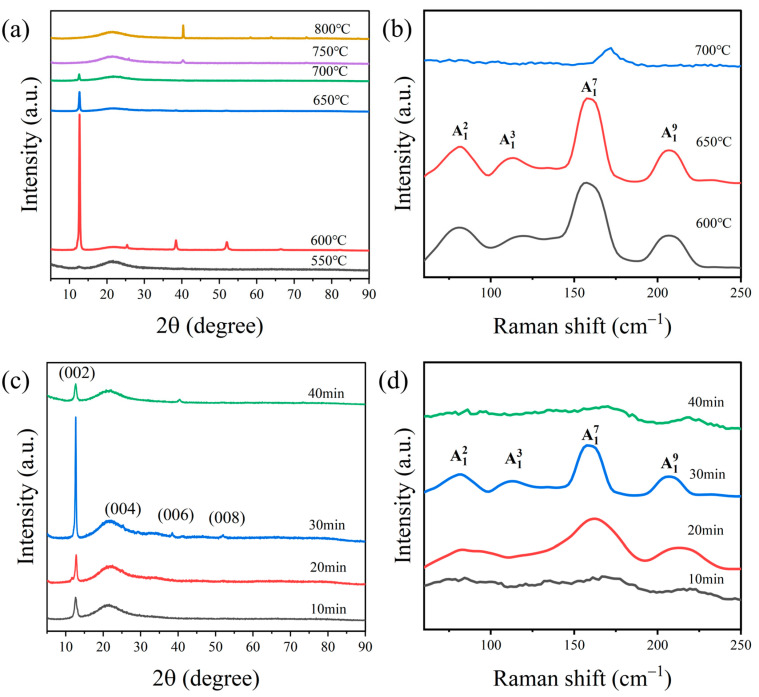
(**a**) XRD patterns of WTe_2_ thin films prepared at different reaction temperatures; (**b**) Raman spectra of WTe_2_ thin films prepared at different reaction temperatures; (**c**) XRD patterns of WTe_2_ thin films prepared at different reaction times; (**d**) Raman spectra of WTe_2_ thin films prepared at different reaction times.

Figure 2a–d show the WTe_2_ prepared under different temperature conditions and reaction times. Figure 2a displays the XRD patterns of tungsten ditelluride films prepared at different temperatures with a reaction time of 30 min. Temperature is a crucial factor that influences the preparation of thin films. Given that the melting point of tellurium is 452 °C, the T2 temperature region was adjusted to 600 °C to guarantee complete melting of the tellurium powder. We primarily studied the impact of temperature variation in the T3 temperature zone, where the tungsten film is located, on the film preparation. We set the reaction temperatures at 550, 600, 650, 700, 750, and 800 °C, respectively. As depicted in the figure, characteristic peaks specific to tungsten ditelluride are observable at temperatures of 700 °C and below. When the temperature is between 750 and 800 °C, the characteristic peak of WTe_2_ does not appear. Instead, the diffraction peak only appears at 2θ = 40.26°, which belongs to the crystalline plane of tungsten (ICSD No.89-3659) (1 1 0). It indicates that the film is still tungsten at 750 and 800 °C. Therefore, when the temperature exceeded 750 °C, WTe_2_ was not generated. This was attributed to the instability of the WTe_2_ film, which is prone to decomposition at high temperatures and reduction to tungsten in a hydrogen atmosphere. The XRD patterns show that the WTe_2_ films generated between 550 and 700 °C have good orientation. The highest XRD peak intensities and higher crystallinity were observed for the samples generated at 600 °C. The XRD data show that the half peak widths of the samples were 0.97423°, 0.28524°, 0.3019°, and 0.3238° under the reaction conditions of a reaction time of 30 min and a reaction temperature of 550–700 °C. It can be concluded that the average grain size of the samples were 8.599, 29.37, 27.7496, and 25.873 nm, respectively. The average particle sizes exhibited an upward trend and then a downward trend with the increase of temperature, and the largest particle size was observed at 600 °C. The strongest diffraction peak at 600 °C is at (0 0 2), and diffraction peaks at (0 0 4), (0 0 6), and (0 0 8) crystal planes appear. This indicates that the crystallinity of the WTe_2_ thin film increases as the reaction temperature rises. However, when the temperature is increased to 600 °C, the intensity of the diffraction peaks gradually decreases until no more tungsten ditelluride films are generated. Therefore, based on the XRD pattern, we determined that the optimum reaction temperature is 600 °C. The three temperatures with the strongest XRD peaks were selected for the Raman spectrum test, and the results are shown in Figure 2b. Four major Raman peaks appeared in the range of 50–250 cm^−1^ at 80.5295, 119.071, 157.449, and 206.549 cm^−1^, which correspond to the W–Te bond $A_{1}^{2}$, $A_{1}^{3}$, $A_{1}^{7}$, and $A_{1}^{9}$ vibrational modes, respectively. The peaks in the Raman spectra at 600 and 650 °C do not differ significantly from each other in terms of their positions and intensities; they all correspond to Raman peaks associated with the Td-phase WTe_2_. At 700 °C, there is only a weak peak at 172.02 cm^−1^ corresponding to the $A_{1}^{7}$ peak. The peak was shifted compared to the $A_{1}^{7}$ peaks at 600 and 650 °C. The rise in temperature caused the evaporation of WTe_2_, resulting in a decrease in the intensity of the peaks in XRD and Raman. From the Raman spectra results, 600 and 650 °C are the optimal preparation temperatures.

Figure 2c shows the XRD patterns of the films prepared at 600 °C with controlled reaction times of 10, 20, 30, and 40 min, respectively. The XRD patterns were compared with the WTe_2_ standard card (ICSD No. 71-2156) and confirmed to be WTe_2_. From the XRD data, it can be seen that the half peak widths of the samples were 0.80419°, 0.75004°, 0.2904°, and 0.662° at a reaction temperature of 600 °C and a reaction time of 10–40 min, respectively. It can be concluded that the average grain sizes of the resulting samples were approximately 10.4175, 11.1696, 28.8485, and 12.655 nm, respectively. The average particle sizes exhibited an upward trend followed by a downward trend over the duration of the reaction, reaching the largest size at 30 min. The (0 0 2) crystal plane diffraction peak was strongest when the reaction time was 30 min. Diffraction peaks corresponding to the (004), (006), and (008) crystal planes were detected in this condition, which were not observed in the 10 and 40 min conditions. This indicates that the films prepared at a reaction time of 30 min have higher crystallinity and more pronounced orientation. Figure 2d shows the Raman spectra at 600 °C for reaction times of 10, 20, 30, and 40 min, respectively. From the figure, it can be seen that the Raman peaks are the most numerous and the strongest signals, and the Raman peaks are the most rounded under the condition of 30 min, which indicates that the signal on the surface of the thin film is stronger, and thus indicates that the concentration of WTe_2_ substance is larger. The peaks of Raman spectra at 10, 20, and 40 min are not obvious, which indicates that the surface structure of the prepared WTe_2_ film is best at the reaction time of 30 min, which is in line with the characterization results of XRD.

**Figure 3:**
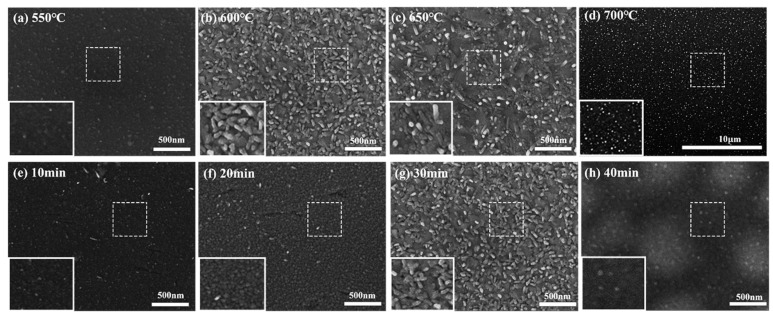
(**a**–**d**) SEM images of WTe_2_ films prepared at different reaction temperatures; (**e**–**h**) SEM images of WTe_2_ films prepared at different reaction times.

To comprehend the variations in WTe_2_ thin film crystals under various conditions and identify the most suitable preparation conditions. We studied the surface morphology of the films using scanning electron microscopy. Figure 3a–d demonstrate the morphological evolution of WTe_2_ films grown at reaction temperatures of 550, 600, 650, and 700 °C for all reaction times of 30 min. Figure 3a is the SEM image at the reaction temperature of 550 °C, and there are a few small particle structures on the surface of the film. Figure 3b shows the SEM image at a reaction temperature of 600 °C. It is evident that there are more rod-like structures on the surface, and they are more uniform, with an average length of about 91 nm. Figure 3c shows the SEM image at a reaction temperature of 650 °C, where the surface displays a rod-like structure of approximately 148 nm. In Figure 3d, taken at a reaction temperature of 700 °C, the surface exhibits a uniformly distributed dot-like structure. The SEM image results show that, as the reaction temperature increased to 600 and 650 °C, the average grain size of the films increased, from the beginning of the aggregation, and formed tiny nuclei later changed to rod-like grains. The length of the rod-like grains at 600 °C was about 91 nm, and the transverse width was about 45 nm. The length of the rod-like grains at 650 °C was about 148 nm, and the transverse width was about 45 nm. The rod-like grains reduced in quantity and became longer in this condition. When the temperature was increased to 700 °C, only a uniformly distributed dot-like structure on the surface was observed. As can be seen from the above, the grain size exhibits first an upward and then a downward trend as temperature rises. The maximum grain size was observed at 650 °C, which aligns with the variation in average grain size with temperature calculated by XRD. This pattern helps us to optimize the growth conditions in order to obtain films with the desired properties. The 600 °C reaction temperature produces a more uniform distribution and the largest number of rod-like grains. Combined with the results of XRD spectroscopy, a temperature of 600 °C favors the growth of grains on the surface of WTe_2_ films, leading to better-oriented WTe_2_ films. 

Figure 3e–h show the morphological evolution of WTe_2_ films grown at a reaction temperature of 600 °C for reaction times of 10, 20, 30, and 40 min, respectively. Figure 3e shows the SEM image at a reaction time of 10 min. The surface of the film exhibits a few rod-like structures and few flakes. Figure 3f shows the SEM image at the reaction time of 20 min. It can be observed that the surface of the film exhibits small particles with a size of approximately 10 nm. Figure 3g shows the SEM image at the reaction time of 30 min. It is evident that there are numerous rod-like structures with a uniform and dense distribution, with an average length of approximately 91 nm. Figure 3h shows the SEM image at a 40 min reaction time, and the surface of the film has fuzzy and small granular structures with poor morphology. Based on the SEM images, the grains growing in the film gradually increase with the reaction time, from small particles with a surface of only 10 nm to rod-like grains with a length of 91 nm and a lateral width of 45 nm. The most numerous and uniform rod-like grains were generated at a 30 min reaction time, but the grain size then became smaller at 40 min. This would be a prolonged hydrogen flux, causing the product to react in other ways. The above results demonstrate that, as the reaction time increases, the grain sizes first become larger and then smaller. The largest and most homogeneous grains are produced at 30 min, aligning with the variation of the average grain size over time as calculated by XRD. This pattern helps us to optimize the growth conditions to obtain films with desired properties. Growth conditions are crucial for obtaining films with desired properties. It also confirms that the uniformly distributed rod-like grains produced under the condition of a 30 min reaction time are more desirable in terms of morphology. Combined with the results of XRD spectroscopy, 30 min is favorable for the growth of WTe_2_ surface grains, which can produce WTe_2_ films with better orientation. 

Combining the XRD, Raman, and SEM characterization results mentioned above, it can be concluded that, as the temperature increases, the films exhibit a noticeable selective orientation and high crystallinity. Additionally, the film’s surface shows uniformly distributed rod-like grains, reaching optimal conditions at 600 °C. Then the crystallinity of the film becomes lower, the orientation is not obvious, and the grains on the surface of the film become smaller until no more tungsten telluride is generated at 750 °C. Therefore, 600 °C is the optimum preparation temperature. The same phenomenon was observed for the reaction time. At 30 min, the WTe_2_ film exhibits a distinct optimal orientation and high crystallinity. The film surface has a uniform distribution of rod-like grains. Therefore, the optimal reaction time of 30 min was determined.

**Figure 4:**
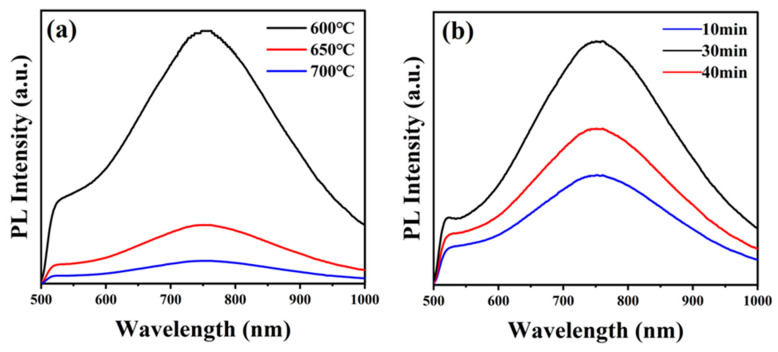
(**a**) Photoluminescence spectra of WTe_2_ films prepared at different temperatures; (**b**) photoluminescence spectra of WTe_2_ films prepared at different reaction times.

To further determine the quality of the samples, luminescence properties were measured. The luminescent properties of WTe_2_ thin films were examined using photoluminescence spectroscopy (PL) with an excitation wavelength of 450 nm. The effects of two factors, reaction temperature and reaction time, on the luminescence properties of WTe_2_ thin films were investigated separately. Figure 4a shows the photoluminescence profiles of the films prepared at various reaction temperatures. It is evident that the fluorescence intensity decreases as the temperature increases, with the highest intensity observed at 600 °C. This indicates that the WTe_2_ films prepared at 600 °C have fewer lattice defects and low photogenerated carrier complexation. Therefore, the films grown under this condition have the strongest luminescence ability. The peak wavelengths and corresponding band gaps achieved for the films prepared at reaction temperatures of 600, 650, and 700 °C are 761.1 nm (1.629 eV), 760.9 nm (1.629 eV), and 760.3 nm (1.631 eV), respectively. The smaller band gap widths achieved at lower temperatures indicate that the WTe_2_ films prepared at reaction temperatures of 600 and 650 °C have a wider range of absorbed photon energies. Figure 4b shows the photoluminescence profiles of the films prepared at different reaction times. It can be observed that the fluorescence intensity is strongest at 30 min. This indicates that the WTe_2_ films prepared with a reaction time of 30 min have few lattice defects and low photogenerated carrier complexation. Hence, the films grown under this condition have the strongest luminescence. The peak wavelengths and corresponding band gaps of the films prepared at reaction times of 10, 30, and 40 min are 760.3 nm (1.631 eV), 761.1 nm (1.629 eV), and 760.3 nm (1.631 eV), respectively. The band gap is minimized at 30 min, which indicates that the WTe_2_ films prepared with a reaction time of 30 min have a larger range of absorbed photon energies. Therefore, the strongest luminescence ability of the films grown at a reaction temperature of 600 °C and a reaction time of 30 min also indicates that the WTe_2_ prepared under this condition has fewer lattice defects and higher crystalline quality.

To evaluate the performance of the samples obtained using this method, we conducted measurements of the samples’ thermal conductivity. The measured samples were tungsten ditelluride films grown at 600 °C for 30 min. The WTe_2_ films grown under these conditions have the fewest defects, enabling a more precise measurement of the material’s thermal conductivity. The practical application of meeting performance and quality requirements provides more reliable data support. The thermal conductivity of WTe_2_ thin films along the C-axis, i.e., in the off-surface direction, at 300 K was tested by laser thermal conductivity.In order to obtain accurate measurements, several measurements were taken on the samples and the results were averaged. The results are shown in Table 1, the thermal conductivities of the three times of the test were 1.175, 1.176, and 1.170 Wm^−1^K^−1^, with an average of 1.173 Wm^−1^K^−1^. The thermal conductivity of few-layer flakes of WTe_2_ (3–20 layers) separated by mechanical stripping and covered on SiO_2_ substrates was 3 Wm^−1^K^−1^, as reported by Mleczko et al. [27]. The minimum thermal conductivity of 30 nm WTe_2_ films prepared by mechanical stripping on 300 nm SiO_2_/Si substrates was 3.0 Wm^−1^K^−1^, as reported by Wei et al. [28]. The in-plane thermal conductivity of 30 nm WTe_2_ films prepared by Zhou et al. using a two-step telluride chemical vapor reaction process was 2 Wm^−1^K^−1^ [29]. The films prepared by our method have lower thermal conductivity compared to the WTe_2_ films prepared by the above methods. This may be due to the fact that the grain size of the films grown under such conditions is smaller, and the heat flow crosses more grain boundaries. Consequently, the resistance to thermal conduction is higher. In addition, grains with irregular orientation and rod-like morphology also increase the scattering of heat flow and decrease the thermal conductivity. Holding other factors constant, the lower the thermal conductivity, the higher the ZT value, which enhances the thermoelectric properties of the film. This indicates that our prepared WTe_2_ thin films are thermoelectric materials with some promising applications [30,31,32,33].

## 4. Conclusions

In this paper, a two-step method is proposed for preparing WTe_2_ thin films. Firstly, the optimal growth conditions for WTe_2_ thin films were determined through parameter modulation. The optimal growth condition was 600 °C, and the reaction time was 30 min. The XRD and Raman tests indicate that the films exhibit clear selective orientation and high crystallinity under these growth conditions. The highest intensity in luminescent properties was also found at a reaction temperature of 600 °C and a reaction time of 30 min. This means that the number of lattice defects in the WTe_2_ films prepared under these conditions is lower. By combining XRD, Raman, XPS, SEM, and photoluminescence tests, we determined that the film morphology with a uniformly distributed rod-like structure resulting from this condition is the most desirable. The rod-like structure grown by this method provides a new idea for growing WTe_2_ nanocrystalline films with low thermal conductivity, and the samples prepared by this method have lower thermal conductivity compared to other methods. This improves the thermal properties of WTe_2_ and increases its applicability in the field of pyroelectricity.

## Figures and Tables

**Table 1:** Thermal conductivity of tungsten telluride films.

	Thermal Diffusion Coefficient (mm^2^/s)	Heat Conductivity (W/m·K)
1	0.789	1.175
2	0.790	1.176
3	0.786	1.170
average value	0.788	1.173

## Data Availability

Data are contained within the article.

## References

[1] Chareev D.A., Evstigneeva P., Phuyal D., Man G.J., Rensmo H., Vasiliev A.N., Abdel-Hafiez M. (2020). Growth of Transition-Metal Dichalcogenides by Solvent Evaporation Technique. Cryst. Growth Des..

[2] Brune V., Grosch M., Weißing R., Hartl F., Frank M., Mishra S., Mathur S. (2021). Influence of the choice of precursors on the synthesis of two-dimensional transition metal dichalcogenides. Dalton Trans..

[3] Wu M., Xiao Y., Zeng Y., Zhou Y., Zeng X., Zhang L., Liao W. (2020). Synthesis of two-dimensional transition metal dichalcogenides for electronics and optoelectronics. InfoMat.

[4] Zhang Z., Yang P., Hong M., Jiang S., Zhao G., Shi J., Xie Q., Zhang Y. (2019). Recent progress in the controlled synthesis of 2D metallic transition metal dichalcogenides. Nanotechnology.

[5] Li Y., Liu J., Zhang P., Zhang J., Xiao N., Yu L., Niu P. (2020). Electrical transport properties of Weyl semimetal WTe_2_ under high pressure. J. Mater. Sci..

[6] Tian W., Yu W., Liu X., Wang Y., Shi J. (2018). A Review of the Characteristics, Synthesis, and Thermodynamics of Type-II Weyl Semimetal WTe_2_. Materials.

[7] Wang Y., Wang L., Liu X., Wu H., Wang P., Yan D., Cheng B., Shi Y., Watanabe K., Taniguchi T. (2019). Direct Evidence for Charge Compensation-Induced Large Magnetoresistance in Thin WTe_2_. Nano Lett..

[8] Liu C., Yan X., Song X., Ding S., Zhang D.W., Zhou P. (2018). A semi-floating gate memory based on van der Waals heterostructures for quasi-non-volatile applications. Nat. Nanotechnol..

[9] Chen Y., Wang X., Huang L., Wang X., Jiang W., Wang Z., Wang P., Wu B., Lin T., Shen H. (2021). Ferroelectric-tuned van der Waals heterojunction with band alignment evolution. Nat. Commun..

[10] Zhou Y., Tong L., Chen Z., Tao L., Li H., Pang Y., Xu J. (2023). Vertical Nonvolatile Schottky-Barrier-Field-Effect Transistor with Self-Gating Semimetal Contact. Adv. Funct. Mater..

[11] Pale P., Mamane V. (2023). Chalcogen Bonding Catalysis: Tellurium, the Last Frontier?. Chem. A Eur. J..

[12] Zhang G., Xu H., Li Y., Xiang C., Ji Q., Liu H., Qu J., Li J. (2019). Interfacial Engineering of SeO Ligands on Tellurium Featuring Synergistic Functionalities of Bond Activation and Chemical States Buffering toward Electrocatalytic Conversion of Nitrogen to Ammonia. Adv. Sci..

[13] Xiong L., Wang K., Li D., Luo X., Weng J., Liu Z., Zhang H. (2020). Research progress on the preparations, characterizations and applications of large scale 2D transition metal dichalcogenides films. FlatChem.

[14] Cao M., Wang Z., Ma L., Zhang L., Wang M., Liu Y., He J., Xi X. (2021). Tungsten Ditelluride: Synthesis, Structure, and Magnetoresistance Property. Adv. Electron. Mater..

[15] Wang X., Wu H., Qiu R., Huang X., Zhang J., Long J., Yao Y., Zhao Y., Zhu Z., Wang J. (2023). Room temperature field-free switching of CoFeB/MgO heterostructure based on large-scale few-layer WTe_2_. Cell Rep. Phys. Sci..

[16] Zhang L., Chen C., Zhou J., Yang G., Wang J., Liu D., Chen Z., Lei W. (2020). Solid Phase Exfoliation for Producing Dispersible Transition Metal Dichalcogenides Nanosheets. Adv. Funct. Mater..

[17] Zhang Y., Wang Z., Feng J., Ming S., Qu F., Xia Y., He M., Hu Z., Wang J. (2022). Synthesis and electromagnetic transport of large-area 2D WTe_2_ thin film. J. Semicond..

[18] Wang Q., Li J., Besbas J., Hsu C.H., Cai K., Yang L., Cheng S., Wu Y., Zhang W., Wang K. (2018). Room-Temperature Nanoseconds Spin Relaxation in WTe_2_ and MoTe2 Thin Films. Adv. Sci..

[19] Mc Manus J.B., Ilhan C., Balsamo B., Downing C., Cullen C.P., Stimpel-Lindner T., Cunningham G., Peters L., Jones L., Mullarkey D. (2020). Synthesis of tungsten ditelluride thin films and highly crystalline nanobelts from pre-deposited reactants. Tungsten.

[20] Giri A., Yang H., Jang W., Kwak J., Thiyagarajan K., Pal M., Lee D., Singh R., Kim C., Cho K. (2018). Synthesis of Atomically Thin Transition Metal Ditelluride Films by Rapid Chemical Transformation in Solution Phase. Chem. Mater..

[21] Asaba T., Wang Y., Li G., Xiang Z., Tinsman C., Chen L., Zhou S., Zhao S., Laleyan D., Li Y. (2018). Magnetic Field Enhanced Superconductivity in Epitaxial Thin Film WTe_2_. Sci. Rep..

[22] Hoang A.T., Qu K., Chen X., Ahn J.H. (2021). Large-area synthesis of transition metal dichalcogenides via CVD and solution-based approaches and their device applications. Nanoscale.

[23] Yang Y., Zhang Y., Yan M. (2022). A review on the preparation of thin-film YSZ electrolyte of SOFCs by magnetron sputtering technology. Sep. Purif. Technol..

[24] Liang J., Liu Q., Li T.S., Luo Y., Lu S., Shi X., Zhang F., Asiri A.M., Sun X. (2021). Magnetron sputtering enabled sustainable synthesis of nanomaterials for energy electrocatalysis. Green Chem..

[25] Ma Y., Li L., Qian J., Qu W., Luo R., Wu F., Chen R. (2021). Materials and structure engineering by magnetron sputtering for advanced lithium batteries. Energy Storage Mater..

[26] Feng L.-P., Jiang W.-Z., Su J., Zhou L.Q., Liu Z.T. (2016). Performance of field-effect transistors based on NbxW1−xS2monolayers. Nanoscale.

[27] Mleczko M.J., Xu R., Okabe K., Kuo H.-H., Fisher I.R., Wong H.-S.P., Nishi Y., Pop E. (2016). High Current Density and Low Thermal Conductivity of Atomically Thin Semimetallic WTe_2_. ACS Nano.

[28] Wei Y., Deng C., Zheng X., Chen Y., Zhang X., Luo W., Zhang Y., Peng G., Liu J., Huang H. (2021). Anisotropic in-plane thermal conductivity for multi-layer WTe_2_. Nano Res..

[29] Zhou Y., Jang H., Woods J.M., Xie Y., Kumaravadivel P., Pan G.A., Liu J., Liu Y., Cahill D.G., Cha J.J. (2017). Direct Synthesis of Large-Scale WTe_2_ Thin Films with Low Thermal Conductivity. Adv. Funct. Mater..

[30] Kanahashi K., Pu J., Takenobu T. (2019). 2D Materials for Large-Area Flexible Thermoelectric Devices. Adv. Energy Mater..

[31] Pan Y., He B., Helm T., Chen D., Schnelle W., Felser C. (2022). Ultrahigh transverse thermoelectric power factor in flexible Weyl semimetal WTe_2_. Nat. Commun..

[32] Pallecchi I., Manca N., Patil B., Pellegrino L., Marre D. (2020). Review on thermoelectric properties of transition metal dichalcogenides. Nano Futur..

[33] Li L., Han W., Pi L., Niu P., Han J., Wang C., Su B., Li H., Xiong J., Bando Y. (2019). Emerging in-plane anisotropic two-dimensional materials. InfoMat.

